# A193 DEMOGRAPHIC, SOCIAL AND OCCUPATIONAL FACTORS THAT PREVENTED EXPOSURE TO SARS-COV-2 IN INFLAMMATORY BOWEL DISEASE PATIENTS DURING THE COVID-19 PANDEMIC: A PROSPECTIVE COHORT STUDY

**DOI:** 10.1093/jcag/gwac036.193

**Published:** 2023-03-07

**Authors:** L N Caplan, N Sharifi, A Markovinovic, M Herauf, J Quan, L Hracs, J W Windsor, S Coward, C Ma, R Panaccione, B Hagel, G G Kaplan

**Affiliations:** 1 Community Health Sciences; 2 IBD Clinic- Department of Medicine, University of Calgary, Calgary, Canada

## Abstract

**Background:**

The COVID-19 pandemic caused by the SARS-CoV-2 virus is a rapidly evolving public health emergency in which mundane behaviors such as grocery shopping or restaurant dining are considered high-risk for some, such as persons with inflammatory bowel disease (IBD) who are often immunodeficient due to medications. Research on the behavioral exposures experienced by populations with IBD during the COVID-19 pandemic are lacking.

**Purpose:**

We aim to better understand how the behaviors of persons with IBD are associated with COVID-19 diagnoses.

**Method:**

We conducted a prospective serosurveillance cohort study in Calgary to assess exposure to SARS-CoV-2 from Nov. 1, 2020 to Aug. 8, 2022 in 485 individuals with IBD. A diagnosis of SARS-CoV-2 was defined as a molecular-confirmed PCR test, a self-report home antigen test, or a positive nucleocapsid antibody level. Participants completed a self-report electronic questionnaire on social and occupational risk activities stratified across two time periods: Jan. 2020 to Mar. 2020 (before lockdown) and post-Jun. 2020 (post lockdown). Univariate analyses (*χ*^2^ and Fischer’s exact if *n*≤5) were performed on social activities that occurred following the lockdown among those with IBD who were and were not diagnosed with COVID-19. Occupational exposures were compared across essential workers (EW) (i.e., frontline workers at high risk of COVID) and non-EWs.

**Result(s):**

Overall, 37.5% (n=182) of our cohort was diagnosed with COVID-19. Seniors were less likely to be infected with COVID-19 (22.7%) compared to those under the age of 65 (40.8%) (p=0.002). A greater proportion of females (42.6 %) compared to males (32.5%) were COVID positive (p=0.02). Those with Crohn’s disease (38.3%) were as likely to test positive for COVID-19 as those with ulcerative colitis (36%) (p=0.65). COVID positive patients were less likely to have 4 vaccine doses (28.5%) compared to those who tested negative (71.5%) (p=0.4).

Statistically significant decreases (p<0.001) in engagement post-Jun. 2020 were observed for: bar use (11.6% to 2.1%), visiting a friend (44.5% to 15.2%), having visitors over (38.7% to 12.1%), restaurant dining (38% to 9%), indoor fitness (31.9% to 8.4%), and transit use (11% to 1.3%). There was an increase in regular use of outdoor fitness (31.9% to 67.1%, p<0.003).

Persons with IBD who tested positive for COVID-19 were more likely to regularly dine in a restaurant (16.8% vs. 4.7% for COVID negative, p<0.001), engage in indoor fitness activities (14% vs. 5.1%, p<0.001), and travel outside Calgary (21% vs. 11.2%, p=0.004) post-lockdown.

Post-lockdown, a greater proportion of EW were COVID positive (50.4%) compared to non-EW (38.6%) (p=0.04).

**Image:**

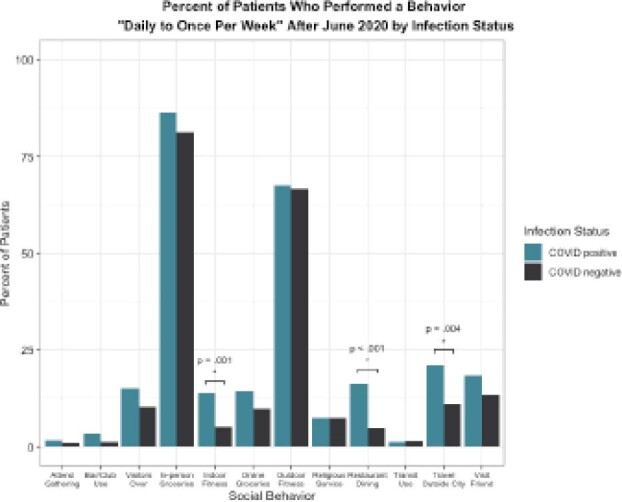

**Conclusion(s):**

Over a two-year period, two-thirds of our cohort did not test positive for COVID-19. Those with IBD who avoided COVID tended to be older, male, have 4 doses of vaccine, and reduce their risk of exposure through social and occupational modifications, perhaps in response to public health guidance.

**Disclosure of Interest:**

None Declared

